# Editorial: Standards in personalized phage therapy: from phage collection to phage production

**DOI:** 10.3389/fcimb.2024.1376386

**Published:** 2024-03-22

**Authors:** Saija Kiljunen, Grégory Resch

**Affiliations:** ^1^ Human Microbiome Research Program, Research Programs Unit, Medicum, Faculty of Medicine, University of Helsinki, Helsinki, Finland; ^2^ Laboratory of Bacteriophages and Phage Therapy, Center for Research and Innovation in Clinical Pharmaceutical Sciences (CRISP), Lausanne University Hospital (CHUV), Lausanne, Switzerland

**Keywords:** bacteriophage, phage, phage therapy, antibiotic resistance (ABR), standardisation

The World Health Organization (WHO) has declared antibiotic resistance to be one of the biggest threats of our time to global health. Phage therapy, the use of viruses that infect bacteria to cure bacterial infections, is one of the solutions that can be used to tackle the problem. Before phage therapy can become a generally acceptable treatment, guidelines concerning the pipeline from phage isolation and characterization to application to patients need to be created and methods applied by different laboratories and companies need to be standardized. The objective of the Research Topic, “*Standards in Personalized Phage Therapy: from phage collection to phage production*,” was to foster discourse about the standards and practices employed in laboratories engaged in the development of personalized phage therapy. The aim was to explore the extent to which standardization can be achieved across various aspects of therapeutic phage production, including phage isolation, characterization, storage, production and purification, and selection.

The Research Topic includes six articles in total: four original research papers and two mini reviews. The published articles cover different aspects of phage therapy research. Two of the papers, original research articles by Štrancar et al. and García-Cruz et al., describe isolation and characterization of phages infecting *Staphylococcus epidermidis* and *Pseudomonas aeruginosa*, both important target bacteria for phage therapy. In addition to the basic phage characterization, the paper by Štrancar et al. discusses how the bacterial host to be used for phage production should be characterized. They suggest that, for the optimal outcome of phage therapy trials, the genomes of strains used for phage production should be sequenced and screened for inducible prophages, toxin genes, and other virulence factors whose presence could lead to contamination of the phage product. The paper by García-Cruz et al. shows how even a slight change in the protein part of a phage tail can alter the phage host range by allowing the phage to utilize a different receptor structure, illustrating the potential of closely related phage variants to extend the host range of therapeutic phage cocktails.

Two of the articles in the Research Topic, the mini review by Daubie et al. and the original research article by Patpatia et al., address phage susceptibility tests (PSTs) from a clinical diagnostic perspective. The mini review article by Daubie et al. reviews used methods for PST and discusses specifications that a routinely used PST method should meet. They conclude that short turnaround time, proper standardization, and easy-to-use technology in PST are critical for phage therapy to become widely implemented. The paper by Patpatia et al. presents a new, liquid-based PST method using hydrogel as phage matrix, allowing phages to be aliquoted to microtiter plate wells up to two months before the assay, thus allowing faster measurement and shorter turnaround time for the assay.

The last two papers in the Research Topic, a mini review by Fujimoto and Uematsu and an original research article by Maimati et al., discuss phage therapy from a slightly wider perspective. The article by Fujimoto and Uematsu summarizes the research addressing phage therapy against *Clostridioides difficile* infection and discusses the difficulties in finding lytic *C. difficile* -specific phages, the possibility of using phage-derived endolysins to control *C. difficile* infection, and the potential of metagenomic data in the identification of novel phage endolysins against non-culturable (intestinal) bacteria. They conclude that the development of more sophisticated methods for phage research will hopefully enable the therapeutic use of phage endolysins against *C. difficile* in the near future. The article by Maimati et al. is a bibliometric analysis of phage therapy research over a 20-year period, from 2001 to 2021. They show the power of bibliometric analysis in gathering data on research trends and show how the emphasis on phage research has shifted from basic research to clinical applications during the study period. They also illustrate how bibliometric data can reveal different emphases in science policy and research funding in different countries and speculate that it can even be used to predict future research trends and hotspots.

We hope that the articles in this Research Topic will pave the way for discussion about standardization and the directions needed for future research to make phage therapy an available treatment method for bacterial infections that are not treatable with chemical antimicrobials alone.

## Author contributions

SK: Writing – original draft. GR: Writing – review & editing.

